# Photoacoustic imaging of rat kidney tissue oxygenation using second near-infrared wavelengths

**DOI:** 10.1117/1.JBO.30.2.026002

**Published:** 2025-02-18

**Authors:** Vinoin Devpaul Vincely, Carolyn L. Bayer

**Affiliations:** Tulane University, Department of Biomedical Engineering, New Orleans, Louisiana, United States

**Keywords:** spectral photoacoustic imaging, blood oxygenation, second near-infrared, spectral coloring, *in vivo*

## Abstract

**Significance:**

Conventionally, spectral photoacoustic imaging (sPAI) to assess tissue oxygenation (${\mathrm{sO}}_{2}$) uses optical wavelengths in the first near-infrared (NIR-I) window. This limits the maximum photoacoustic imaging depth due to the high spectral coloring of biological tissues and has been a major barrier to the clinical translation of the technique.

**Aim:**

We demonstrate the second near-infrared (NIR-II) tissue optical window (950 to 1400 nm) for the assessment of blood and tissue ${\mathrm{sO}}_{2}$.

**Approach:**

The NIR-II PA spectra of oxygenated and deoxygenated hemoglobin were first characterized using a phantom. Optimal wavelengths to minimize spectral coloring were identified. The resulting NIR-II PA imaging methods were then validated *in vivo* by measuring kidney ${\mathrm{sO}}_{2}$ in adult female rats.

**Results:**

sPAI of whole blood, in a phantom, and of blood in kidneys *in vivo* produced PA spectra proportional to wavelength-dependent optical absorption. Using the NIR-II wavelengths for spectral unmixing resulted in a ∼50% decrease in the error of the estimated blood ${\mathrm{sO}}_{2}$, compared with conventional NIR-I wavelengths. *In vivo* measurements of kidney ${\mathrm{sO}}_{2}$ validated these findings, with a similar 50% reduction in error when using NIR-II wavelengths versus NIR-I wavelengths at larger illumination depths.

**Conclusions:**

sPAI using NIR-II wavelengths improved the accuracy of tissue ${\mathrm{sO}}_{2}$ measurements. This is likely due to reduced scattering, which reduces the attenuation and, therefore, the impact of spectral coloring in this wavelength range. Combined with the increased safe skin exposure fluence limits in this wavelength range, these results demonstrate the potential to use NIR-II wavelengths for quantitative sPAI of ${\mathrm{sO}}_{2}$ from deep heterogeneous tissues.

## 1 Introduction

Oxygen saturation of hemoglobin (${\mathrm{sO}}_{2}$) is an important physiological metric for clinical applications in oncology,^1^^–^^3^ neurology,^4^^–^^7^ cardiology,^8^ and obstetrics.^9^^–^^11^ Spectral photoacoustic imaging (sPAI) is a promising method to measure deep tissue ${\mathrm{sO}}_{2}$ due to its non-invasive and non-ionizing nature while providing images in real time. sPAI uses optical excitation to generate broadband acoustic waves through thermoelastic expansion—a rapid conversion of optical to mechanical energy that occurs upon absorption of light by chromophores. In contrast to purely optical imaging techniques, PAI allows visualization of deeper structures due to the reduced sound attenuation by biological tissues.

Photoacoustic (PA) image contrast is directly proportional to the local optical fluence and the concentration of optical chromophores, including oxyhemoglobin (oxyHb) and deoxyhemoglobin (deoxyHb), within the imaged region. sPAI leverages the distinct optical spectral features of oxyHb and deoxyHb by acquiring multiple images at different wavelengths to estimate their spatially varying concentration within the imaged tissue, typically through linear spectral unmixing of the measured photoacoustic signals. One of the earliest demonstrations of measuring blood ${\mathrm{sO}}_{2}$ using sPAI was performed by Wang et al.^12^ to monitor cerebral vasculature in small-animal models, with imaging performed between 580 and 600 nm. sPAI has been extended to various biomedical applications—Diot et. al. performed blood ${\mathrm{sO}}_{2}$ up to depths of 2.2 cm in breasts containing invasive lobular carcinoma using excitations between 700 and 970 nm.^13^ More recently, our group measured placental oxygenation in normal pregnant and preeclamptic rats with optical excitations at 690, 808, and 950 nm, up to a depth of 2 cm.^10^ Conventionally, sPAI for measuring ${\mathrm{sO}}_{2}$ has been implemented using wavelengths in the first near-infrared (NIR-I) window (690 to 950 nm).^9^^,^^10^^,^^12^ However, high spectral coloring within this optical window limits the accuracy of quantitative measurements at larger *in vivo* depths (beyond ∼1  cm). The accuracy of the sPAI ${\mathrm{sO}}_{2}$ measurements is affected by the wavelength- and spatially variant local fluence at the imaged target. Photons undergo a complex wavelength-dependent attenuation as they propagate through heterogeneous biological tissues due to the spectrally and spatially varying optical properties (absorption and scattering) of different tissue types. This results in distortions in the measured PA spectrum of the imaged targets, termed spectral coloring. Various studies have attempted to account for the spectrally varying local fluence.^14^^–^^19^ Optical transport models, such as Monte Carlo (MC) simulations, have been widely used to model the light distribution in tissues to account for the spatially varying local fluence and improve the accuracy of estimation of blood oxygenation.^14^^–^^17^ However, accurate simulations require prior knowledge of the optical properties of the heterogeneous tissues within the imaged volume. Direct measurements of local fluence using diffuse optical tomography (DOT) have also been demonstrated.^18^^,^^19^ However, besides requiring additional imaging hardware, DOT has insufficient spatial accuracy to correct tissue optical properties adequately.

sPAI using second near-infrared (NIR-II) wavelengths (950 to 1800 nm) offers advantages, including exponentially reduced optical scattering by biological tissues and higher maximum safety exposure limits (MPE) for skin,^20^ potentially facilitating deep tissue sPAI of ${\mathrm{sO}}_{2}$. PA imaging within the NIR-II often uses contrast agents that absorb strongly within this optical window.^20^^–^^24^ Chitgupi et al.^25^ demonstrated imaging up to depths of 12 cm under chicken breast using NIR-II micelles. Recently, our group showed a biocompatible NIR-II semiconductor nanocrystal to image up to ∼6.5  cm in heterogenous porcine tissue.^23^ NIR-II wavelengths have been widely employed for imaging collagen and lipids^26^^–^^31^ for label-free imaging due to their strong optical absorption within this window.^24^ Kruizinga et al.^31^ used NIR-II wavelengths to assess the presence of atherosclerotic (lipid-rich) lesions. Various studies have demonstrated anatomical visualization of hemoglobin-carrying vasculature.^32^^–^^34^ For example, Lin et al.^33^ demonstrated imaging of deeply embedded vasculature (∼4  cm) in a human breast using 1064 nm. However, the use of the NIR-II window for sPAI-derived measurements of ${\mathrm{sO}}_{2}$ has yet to be demonstrated. In this study, we assessed spectral unmixing of hemoglobin oxygenation using NIR-II wavelengths in a porcine phantom and validated these results by measuring renal tissue oxygenation in rats.

## 2 Methods

### 2.1 Photoacoustic Imaging Instrumentation

PA imaging was performed using a pulsed optical parametric oscillator laser (Phocus, BENCHTOP, Opotek Inc., Carlsbad, California, United States) integrated with an open architecture data acquisition system (Vantage 256, Verasonics Inc., Kirkland, Washington, United States), using a 6-MHz linear array transducer (L7-4, Philips, Amsterdam, Netherlands). The laser generates 5-ns pulses at a 10-Hz repetition rate at tunable excitation wavelengths in the NIR-I (690 to 950 nm) and NIR-II (1064, 1200 to 2400 nm) at 1-nm increments. The optical energy incident on the surface of the imaged target was measured using a power meter (Ophir Technologies, West North Logan, Utah, United States) and maintained at all wavelengths with an average pulse-to-pulse variation in laser output of less than 5%. Burn paper was used to measure the beam diameter because the beam diameter affects light penetration depth.^35^^–^^37^ All data analysis was performed in MATLAB.

### 2.2 Porcine Phantom for NIR-II Blood Characterization

**Fig. 1:**
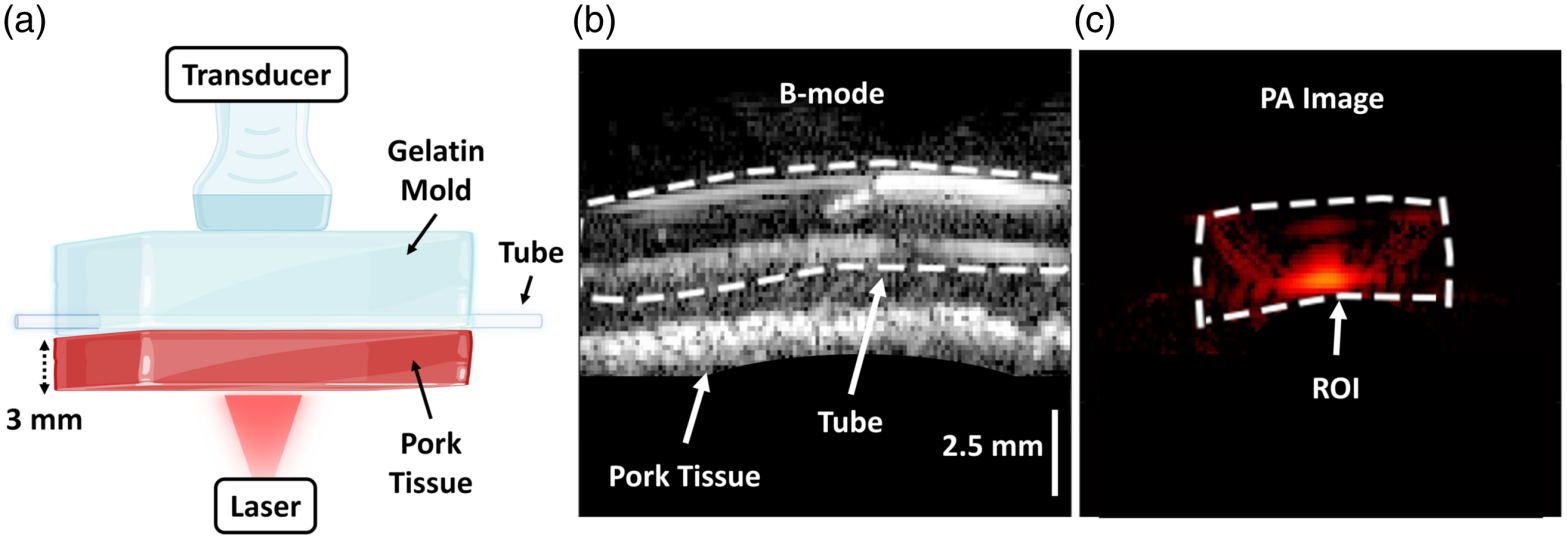
Porcine tissue phantom for NIR-II characterization of blood oxygenation. (a) A schematic representation of the porcine tissue phantom used to characterize the NIR-II PA spectra of oxygenated and deoxygenated blood. (b) B-mode image of a 3-mm slice of pork with a tube carrying blood underneath embedded in gelatin (scale bar = 2.5 mm). (c) PA image of the tube carrying oxygenated blood acquired using 1064-nm laser illumination.

A freshly cut slice of porcine muscle with thicknesses of 3 mm (providing an optical path length of 0.3 cm) was thoroughly washed and placed over a polytetrafluoroethylene tube (1.5/2.0 mm inner/outer diameter) embedded in a gelatin mold [as depicted in Fig. 1(a)]. The transducer array was placed antiparallel to the illumination surface to ensure reproducible air-beam alignment. The pink coloration of pork is due to myoglobin, with a low absorption in the NIR-II window.^38^ Minimal blood remains in muscle tissue after butchering and preparation; therefore, the tissue was assumed to comprise lipids and water as the only optical absorbers. Defibrinated whole bovine blood (910-100, Quad Five, Ryegate, Montana, United States) at two levels of oxygenation—100% and 0%, and a phosphate-buffer solution (PBS) were loaded in the tube sequentially. Deoxygenation of the blood was performed using sodium hydrosulfite (${\mathrm{Na}}_{2}S_{2}O_{4}$, 157953-100G, Sigma-Aldrich, St. Louis, Missouri, United States) at concentrations where conversion to methemoglobin is limited.^39^ The stability of the targeted ${\mathrm{sO}}_{2}$ was monitored using a bare-fiber oxygen sensor coupled with an OxyLite system (Oxford Optronix, Oxford, United Kingdom) at regular intervals throughout imaging. PA images were collected at NIR-I (690 to 950 nm at 20-nm increments) and NIR-II (1064, 1200 to 1400 nm at 10-nm increments) using a $25\;\mathrm{mJ}/{\mathrm{cm}}^{2}$ surface fluence and a circular beam with a diameter of 0.5 cm. The phantom experiments were repeated three times (i.e., three separately prepared phantoms).

In the collected PA images, a region of interest (ROI) was selected around the tube, and the pixels within this region were averaged. The measured PA spectra were normalized to the isosbestic point of hemoglobin (808 nm) to account for measurement variations due to tissue inhomogeneity across different experimental runs. Next, the local fluence at the tube within the phantom was modeled using MC simulations (details provided in Fig. S1 in the Supplementary Material). The calculated local fluence was adjusted based on the fluence at the illumination surface as measured using the power meter. This was then used to normalize the raw measurements and obtain the final PA spectra of oxygenated and deoxygenated blood.

### 2.3 *In Vivo* NIR-II Imaging of Kidney Vasculature in Rat

**Fig. 2:**
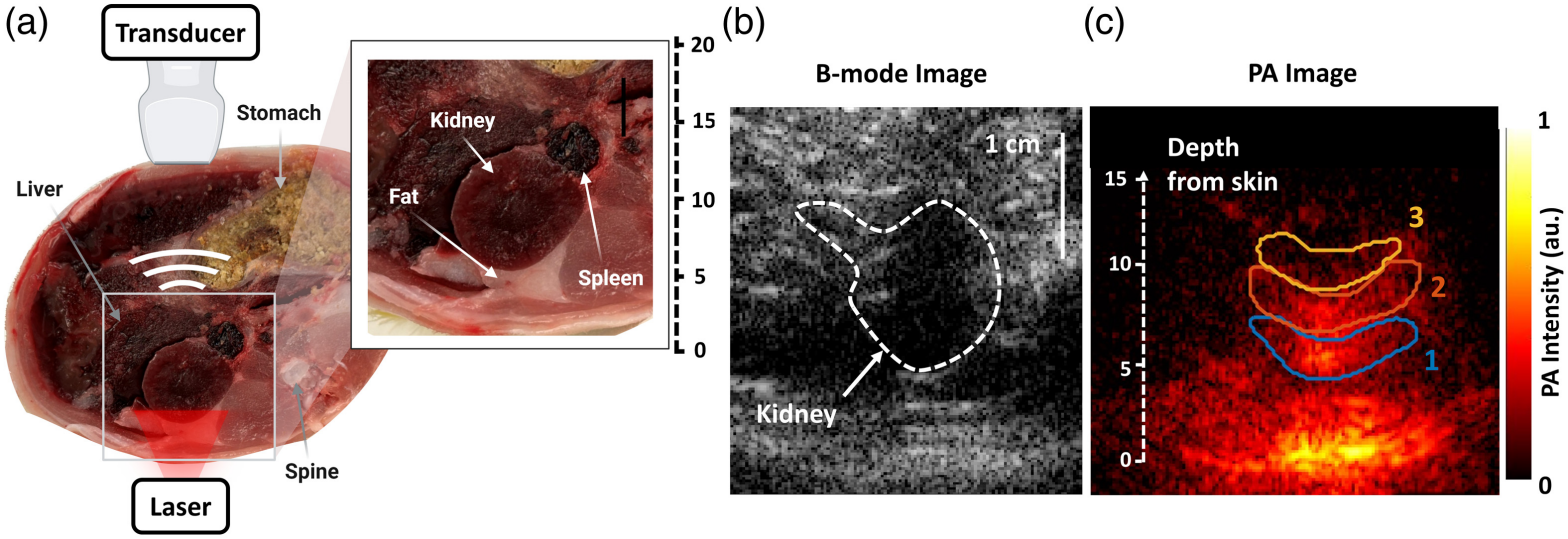
In vivo NIR-II PA imaging of rat kidney. (a) A photograph of a cross-section of the female rat abdomen with a description of the orientations of the light delivery and the acoustic detection for imaging the kidney. The inset replicates the 2D slice of the rat abdomen acquired with the PA system. (b) A B-mode image of the abdomen with a cross-section of the kidney (indicated with an arrow). (c) PA image of the rat kidney collected at 1064 nm. Three unique ROIs are delineated within the kidney at varying depths from the surface (blue contour: ∼6  mm, orange contour: ∼9  mm, and yellow contour: ∼12  mm).

All animal imaging and surgical procedures followed protocols approved by the Institutional Animal Care and Use Committee (IACUC) at Tulane University. Adult female Sprague Dawley rats (n=3) were acquired from a commercial vendor (Charles River Laboratories, Boston, Massachusetts, United States). The animals were anesthetized using 3% isoflurane, and the hair on the abdomen and back was removed using a depilatory cream. The animal was then moved to a transparent sheet (polyethylene terephthalate) for illumination of the posterior side of the animal [as displayed in Fig. 2(a)]. A circular beam of 1.3-cm diameter was used to deliver an optical fluence of 5 to $6\;\mathrm{mJ}/{\mathrm{cm}}^{2}$ at the surface of the animal’s skin (accounting for energy attenuation by the transparent sheet, Fig. S2 in the Supplementary Material). The 1.3-cm beam fluence could not be increased further due to limited energy from the laser, except at 1064 nm, as this is the fundamental frequency of the laser. The breathing rate and temperature of the animal’s skin were monitored during the experiment. The 6-MHz linear transducer was placed on the abdomen and positioned over the animal’s kidney. PA images (five frames per image) were acquired using NIR-I (690 to 950 nm with 20-nm increments) and NIR-II (1064, 1200 to 1300 nm with 10-nm increments). After the imaging session, the oxygenation of the kidney was measured over 10 min using a surgically inserted oxygen probe. The animal was euthanized using excess ${\mathrm{CO}}_{2}$ for a total of 15 to 20 min, followed again by PA imaging. The kidney oxygenation of the euthanized rat was again measured after imaging, ∼1.5  h post-euthanasia, using the surgically inserted oxygen probe. To demonstrate that the measured PA signal was primarily generated by hemoglobin, the kidney of a euthanized animal was removed and bleached of hemoglobin by suspending the tissue in excess hydrogen peroxide overnight (∼15  h).^25^^,^^26^ The bleached kidney was then placed back in the abdomen of the euthanized animal and imaged.

To characterize the PA spectra of the vasculature in the kidney under different conditions (pre- and post-euthanasia and bleached of hemoglobin), three unique ROIs within the kidney were delineated at varying depths from the surface of illumination, and the pixels within the region were averaged [Fig. 2(c)]. An ROI around the entire kidney (indicated with a white outline on the B-mode image) was used to average signal from the whole kidney [Fig. 2(b)]. On one animal, a characteristic artifact, caused by an out-of-plane bubble, was segmented out of the ROI before further analysis. The PA signal was further averaged across the five acquired frames. To account for PA signal variation due to motion artifacts and variations in animal size, the spectral PA images were normalized at the isosbestic point (808 nm). In the *in vivo* model, light propagated through a layer of skin and subcutaneous fat before illuminating the kidney [as seen in the inset of Fig. 2(a)]. The light attenuation by these different tissue layers was accounted for by modeling the light distribution using Monte Carlo simulations (detailed methods described in Fig. S3 in the Supplementary Material).^40^ A layered mask based on Fig. 2(a) (inset) was developed, and the optical properties of each layer were set to values reported in the literature.^41^^,^^42^ A power analysis of the total absolute error was performed using G*Power Software (Heinrich-Heine-Universität Düsseldorf, Düsseldorf, Germany). A total power of ∼>0.99 was observed at regions 2 and 3 for a sample size of 3 (α=0.01), whereas region 1 was not sufficiently powered to perform statistical analysis. A one-way ANOVA was performed, and the p-values indicated significant differences between estimates of ${\mathrm{sO}}_{2}$ in the NIR-I and NIR-II for regions 2 and 3 [Fig. 4(d)].

### 2.4 Spectral Unmixing of Blood Oxygenation

The averaged PA signals, $p_{0}(\lambda )$, were linearly unmixed to estimate the concentrations of oxyHb $c_{{\mathrm{HbO}}_{2}}$, and deoxyHb, $c_{\mathrm{Hb}}$, within the imaged target using Eq. (1)

$$p_{0}(\lambda )=\Gamma [\epsilon_{{\mathrm{HbO}}_{2}}(\lambda )c_{{\mathrm{HbO}}_{2}}+\epsilon_{\mathrm{Hb}}(\lambda )c_{\mathrm{Hb}}]\Phi (\mu_{a},\mu_{s},\lambda ).$$ (1)

where $\epsilon_{i}$ is the molar optical extinction coefficient of the target chromophore—in this case, either oxyHb or deoxyHb, as a function of the illumination wavelength (λ), and Φ represents the local optical fluence, which is a function of both the wavelength and the optical properties of the ambient medium. Molar extinction values are retrieved from.^43^ $c_{i}$ is the distribution of the target chromophores. Once the oxyHb and deoxyHb concentrations are estimated, blood oxygenation, ${\mathrm{sO}}_{2}$, can be calculated using Eq. (2)

$${\mathrm{sO}}_{2}=\frac{c_{{\mathrm{HbO}}_{2}}}{c_{{\mathrm{HbO}}_{2}}+c_{\mathrm{Hb}}}.$$ (2)

## 3. Results

### 3.1 NIR-II PA Characterization of Blood Oxygenation

**Fig. 3:**
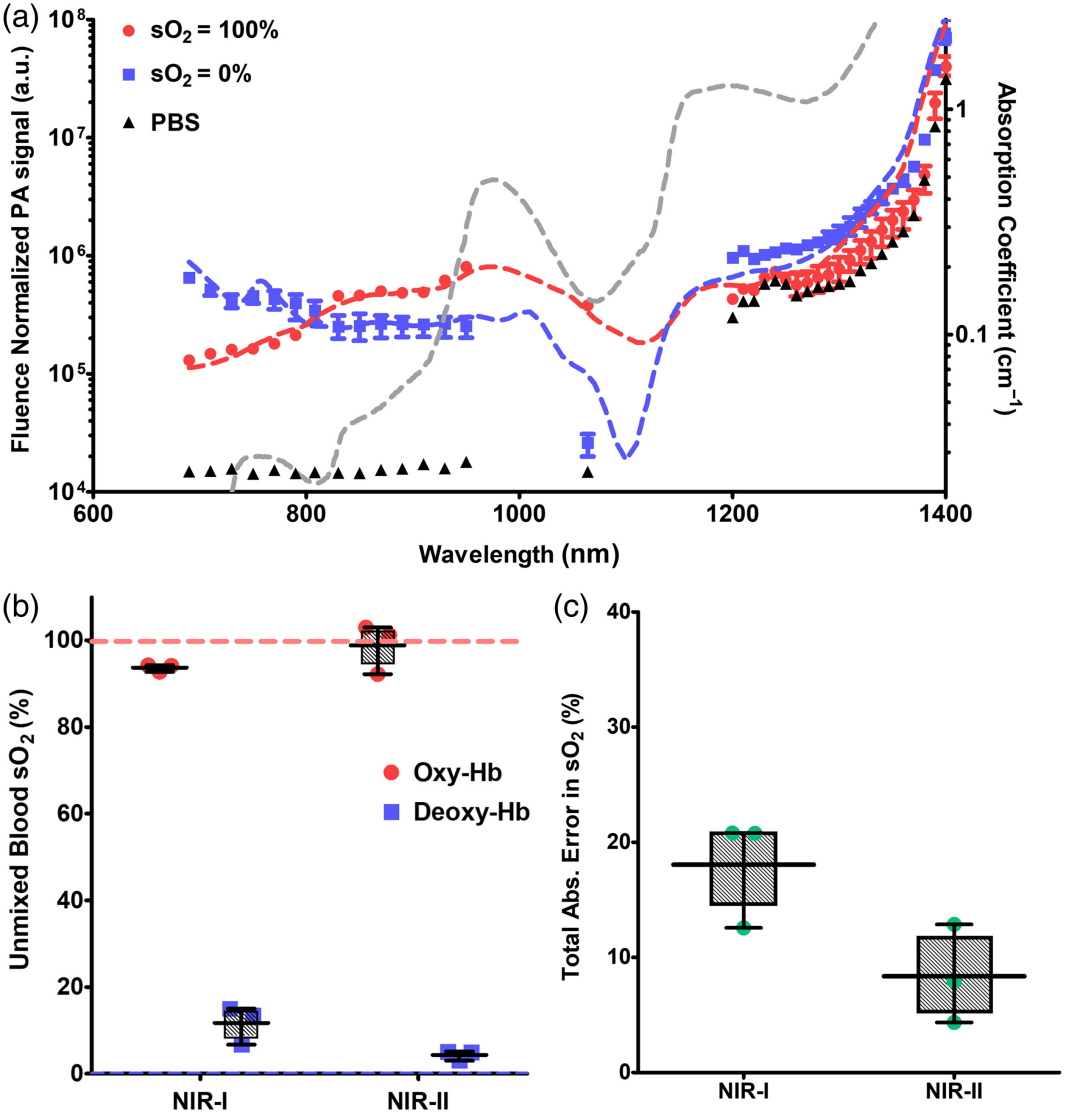
Spectral photoacoustic signal from whole blood in a porcine phantom. (a) A plot of normalized PA spectra from oxygenated blood (red circles), deoxygenated blood (blue squares), and PBS (black triangles) from NIR-I and NIR-II windows. These PA data were acquired using a fluence of $25\;\mathrm{mJ}/{\mathrm{cm}}^{2}$ at the pork surface and a circular beam of 0.5-cm diameter. Dashed lines represent the literature values (derived from Ref. 25) of optical absorption of oxyHb (red), deoxyHb (blue), and water (gray) within this spectral window. (b) A box plot of spectrally unmixed blood ${\mathrm{sO}}_{2}$ using a combination of NIR-I (690, 808, and 950 nm) and NIR-II (1064 and 1230 nm) wavelengths. Colored dashed lines represent the true ${\mathrm{sO}}_{2}$ of the blood in the tube. (c) A boxplot of the total absolute error (linear sum of absolute errors in oxygenated and deoxygenated blood) in estimated ${\mathrm{sO}}_{2}$ using NIR-I and NIR-II wavelengths for all three phantom imaging runs. For all boxplots, the upper and lower limits of each box correspond to the 75th and 25th quartiles. The line through the center corresponds to the mean, whereas the whiskers correspond to the range of the data.

A porcine tissue phantom with an embedded sample tube was used to characterize the PA spectra of the oxygenated and deoxygenated blood over a wide spectral range. Figure 3(a) shows the PA spectra of three target solutions (i.e., oxyHb, deoxyHb, and PBS) from 690 to 1400 nm (an extended spectral range up to 1800 nm is included in Fig. S4 in the Supplementary Material). In general, the fluence-normalized PA spectra of oxygenated and deoxygenated blood were consistent with the spectral shape of the optical absorption spectra (from the literature^43^). Beyond 1300 nm, oxygenated blood and PBS are indistinguishable likely due to the similar optical absorption of these species in this wavelength range. Beyond 1300 nm, there is also an exponential rise in the optical absorption of water (which comprises 70% of typical biological tissues), with diminishing differences in optical absorption between the three species (Fig. S4 in the Supplementary Material). However, we noticed a distinction between the three species at 1064 nm and a noticeable difference at 1200 to 1300 nm, indicating that using wavelengths between 950 and 1300 nm may be sufficient for quantitative PA measurements.

Spectral unmixing to estimate blood oxygenation was performed using different combinations of wavelengths. Figure 3(b) shows a bar chart of the blood ${\mathrm{sO}}_{2}$, estimated using spectral unmixing, for two different combinations of laser wavelengths—NIR-I (690, 808, and 950 nm) and NIR-II (1064 and 1230 nm). These specific NIR-II wavelengths were chosen through qualitative assessment of the NIR-II optical absorption spectra as they demonstrate a distinction between oxygenation and deoxygenated hemoglobin, whereas absorption by other chromophores, such as lipids, collagen, and water, remains relatively low.^24^ The total absolute error in the ${\mathrm{sO}}_{2}$ estimation (i.e., a sum of the absolute error in estimated ${\mathrm{sO}}_{2}$ of oxygenated and deoxygenated blood versus probe measurements) using these wavelength combinations is shown in Fig. 3(c). The traditional NIR-I wavelengths used for spectral unmixing had a higher error (∼15% to 20%), whereas NIR-II wavelengths had absolute errors under 10%.

### 3.2 *In Vivo* Validation of sPAI Using NIR-II Wavelengths

**Fig. 4:**
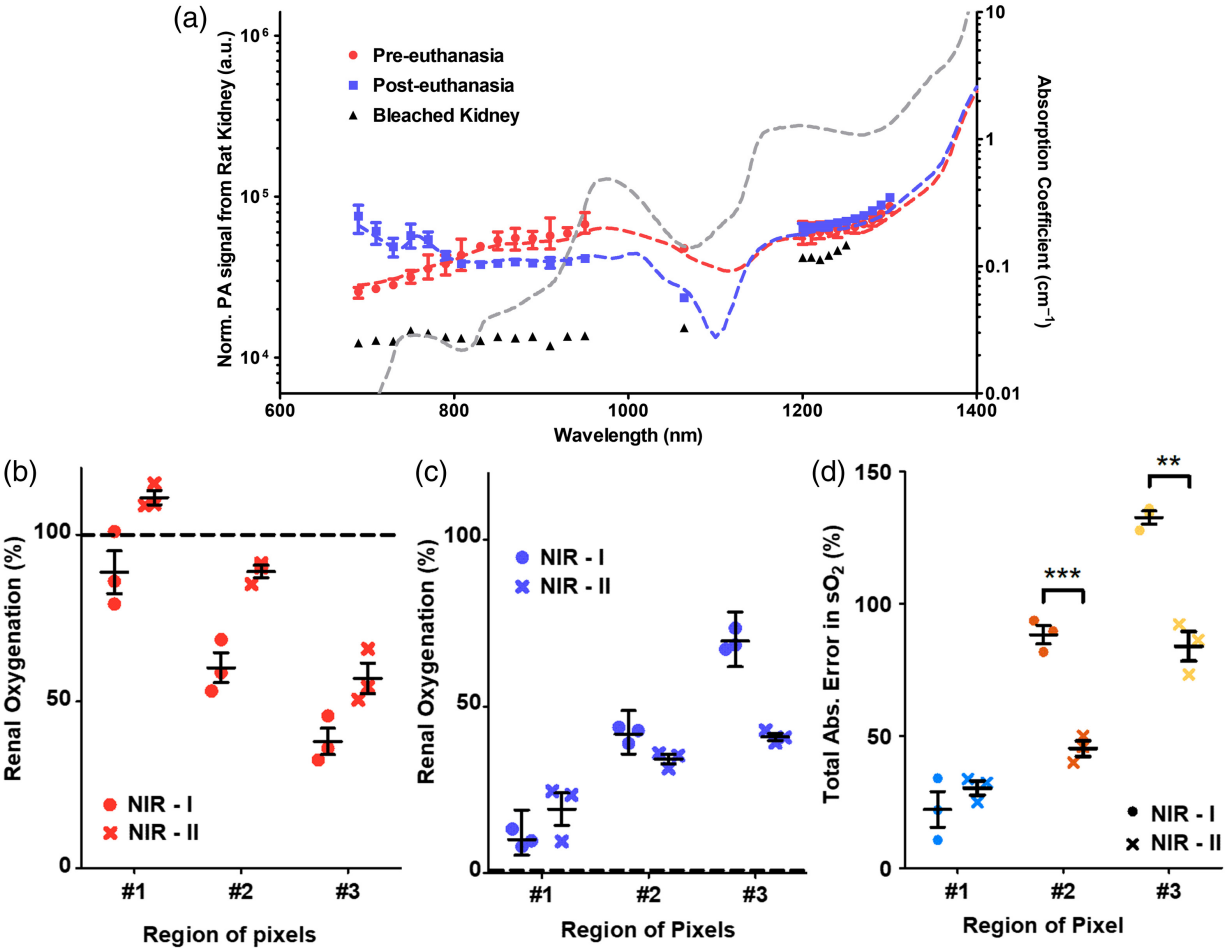
Spectral PA imaging of renal oxygenation using NIR wavelengths. (a) A plot of absorption normalized PA spectra of rat kidney imaged pre- (red) and post- (blue) euthanasia along with a rat kidney bleached of hemoglobin (black). These PA data were acquired using a fluence of 5 to $6\;\mathrm{mJ}/{\mathrm{cm}}^{2}$ at the skin surface and a circular beam of 1.3-cm diameter. The optical absorption spectra of oxyHb (red dashed lines), deoxyHb (blue dashed lines), and water (black dashed lines) are overlayed on the plot. (b), (c) Plots of the renal oxygenation of pre- (b) and post-euthanized (c) rats estimated using spectral unmixing with combinations of wavelengths—NIR-I (690, 808, and 950 nm) and NIR-II (1064 and 1230 nm) within the selected ROIs, bars plotted to indicate the mean, 75th and 25th quartiles (n=3). The true renal oxygenation, measured with the oxygen probe, is indicated in dashed lines. (d) A plot of the total absolute error in renal oxygenation (sum of the absolute error in pre and post-euthanized animal versus probe measurements) within the different depth ROIs, for the two combinations of wavelengths used for spectral unmixing. p-Values from a one-way ANOVA indicated by ***p-value<0.001 and **p-value<0.01.

To validate the phantom experimental results, sPAI of *in vivo* renal ${\mathrm{sO}}_{2}$ was performed using wavelengths from the NIR-I and NIR-II windows. Figure 4(a) shows rat kidneys’ absorption normalized PA spectra under the three unique conditions (pre- and post-euthanasia and bleached of hemoglobin) within the 690- to 1300-nm spectral range. The normalized spectra of the PA signal and the optical absorption spectra show consistent trends between the different hemoglobin species. The PA spectra from oxygenated and deoxygenated kidneys were distinctly higher than from the kidney bleached of hemoglobin, confirming that the measured PA signals are generated by hemoglobin in the kidney. The highest contrast in the spectra between oxygenated and deoxygenated kidneys (∼2× difference in SNR) is at 1064 nm. Furthermore, imaging at the increased fluence available with NIR-II wavelengths results in a 40% increase in the SNR of deeply embedded vasculature (Fig. S5 in the Supplementary Material).

Renal ${\mathrm{sO}}_{2}$ was calculated using both the NIR-I and NIR-II wavelength ranges. Figure 4(b) shows a boxplot of the renal ${\mathrm{sO}}_{2}$ obtained using NIR-I (690, 808, and 950 nm) and NIR-II (1064 and 1230 nm). Consistent with the phantom experiments, the PA spectral features follow the optical absorption spectra of each hemoglobin species. Two different oxygenation levels of the kidney—100% and 0%—were measured using an oxygen probe. The total absolute error in renal ${\mathrm{sO}}_{2}$ estimation (i.e., a sum of the absolute error in sPA-derived kidney ${\mathrm{sO}}_{2}$ compared with the oxygen probe measurements) is shown in Fig. 4(d). Using NIR-I wavelengths, there was an average spectral unmixing error of 22% in region 1, with increased error in deeper areas (88% and 132% for regions 2 and 3, respectively) [Fig. 4(d)]. Using NIR-II wavelengths, regions 2 and 3 demonstrated average errors of 45% and 84%, respectively, an up to 50% reduction in the spectral unmixing errors compared with NIR-I wavelengths.

## 4 Discussion

The primary objective of this work was to demonstrate spectral PA imaging of tissue ${\mathrm{sO}}_{2}$ using NIR-II wavelengths. To achieve this, the PA spectra of oxyHb and deoxyHb within whole blood were first characterized using a phantom (at a wide spectral range of 690 to 1800 nm), and the accuracy of spectral unmixing to estimate blood ${\mathrm{sO}}_{2}$ was assessed. Next, NIR-II photoacoustic imaging performance was validated *in vivo* by imaging the kidneys of adult female rats.

Two unique advantages facilitate deep tissue sPAI using NIR-II wavelengths—the reduced tissue scattering and increased MPE for skin in this wavelength range. Tissue scattering is a key limitation in light penetration depth. Reduced tissue scattering in the NIR-II leads to improved light penetration and visualization of deeper structures. Various studies have demonstrated improved photon penetration using NIR-II wavelengths to minimize tissue scattering for other optical-based imaging modalities.^44^^–^^47^ Zhang et al.^44^ demonstrated high tissue transparency within NIR-II spectral windows using hyperspectral optical imaging, improving photon penetration and spatial contrast. The use of NIR-II wavelengths for fluorescence imaging is a research area currently undergoing rapid development as the reduced tissue scattering enables higher spatial resolution and higher contrast *in vivo*.^46^ Using fluorescence imaging, Byrd et al.^45^ demonstrated a 50% increase in the ratio of vessel-to-tissue fluorescence signal collected at NIR-II over NIR-I wavelengths from an adult pig brain. Similarly, deep tissue anatomical PA imaging using NIR-II wavelengths has been demonstrated.^34^^,^^48^^–^^51^ Recently, Lin et al.^33^ demonstrated high light penetration (up to 4 cm) using 1064-nm illumination allowing PA imaging of deeply embedded breast vasculature in humans. However, these studies were limited to single-wavelength anatomical imaging with no quantitative estimations of blood oxygenation through sPAI. A systematic comparison of NIR-I against NIR-II wavelengths for sPAI of blood oxygenation had yet to be performed. In our studies, a 50% reduction in the error of blood oxygenation estimation was observed in phantoms [Fig. 3(c)] and *in vivo* at larger depths [Fig. 4(d)].

Our results show improved accuracy in the photoacoustic measurement of blood and tissue ${\mathrm{sO}}_{2}$ with the use of NIR-II compared with traditional NIR-I wavelengths. This improved accuracy may be attributed to reduced spectral coloring because of minimized tissue scattering in the NIR-II versus NIR-I (particularly between 1200 and 1300 nm).^52^ Porcine tissue has a total optical attenuation (absorption and scattering of water and lipids) of $∼50\;{\mathrm{cm}}^{-1}$ in the NIR-I window, dominated by diffused photon scattering. By contrast, tissue attenuation in the NIR-II window is under $∼10\;{\mathrm{cm}}^{-1}$ (a 5× decrease).^53^ The accuracy in the estimation of chromophore distribution depends on accurate estimates of local fluence within the tissue. For NIR-II wavelengths, the reduced tissue scattering has a more significant impact on tissue penetration despite the increase in absorption. To illustrate the greater effect of scattering versus absorption in the NIR-II, we performed MCX simulations with varying scattering and absorption values. Our results indicate a 10× increase in fluence when simulating tissue properties at 1064 nm versus 690 nm (Fig. S6 in the Supplementary Material).

In addition, the increased MPE thresholds at NIR-II wavelengths ($100\;\mathrm{mJ}/{\mathrm{cm}}^{2}$ at wavelengths greater than 1050 nm versus $20\;\mathrm{mJ}/{\mathrm{cm}}^{2}$ at 690 nm) will allow higher fluences to be delivered to the imaged target, thereby increasing the imaging depth and image contrast. In our experiments, increasing fluence of the 1064 nm beam by 36% ($36\;\mathrm{mJ}/{\mathrm{cm}}^{2}$ versus $6\;\mathrm{mJ}/{\mathrm{cm}}^{2}$) increased the PA signal from the *in vivo* kidney by ∼40% (Fig. S5 in the Supplementary Material); this supports the potential of extending *in vivo* imaging depth by increasing fluence to the higher MPE using NIR-II wavelengths.

## 5 Conclusions

Our experimental results demonstrate sPAI imaging of tissue ${\mathrm{sO}}_{2}$ using wavelengths in the NIR-II window. We demonstrate improvement in the accuracy of spectral unmixing using NIR-II wavelengths in *ex vivo* porcine tissue phantoms and *in vivo* kidney imaging. We attribute this improvement to the reduced spectral coloring due to reduced tissue scattering when using NIR-II wavelengths. We demonstrated these results up to a depth of ∼1.5  cm *in vivo* at a low fluence of 5 to $6\;\mathrm{mJ}/{\mathrm{cm}}^{2}$ (5% of MPE). Future studies will explore the maximum possible imaging depth in larger animal models and at higher fluences. These results demonstrate the potential for future clinical translation of photoacoustic imaging in the NIR-II to accurately estimate deep tissue oxygenation.

## Supplementary Material

10.1117/1.JBO.30.2.026002.s01

## Data Availability

The code used for spectral unmixing is available on GitHub,^54^ and the fluence-normalized PA data from the *in vivo* and phantom experiments are available on Dryad.^55^
