# Supplementary material for: Photoacoustic imaging of rat kidney tissue oxygenation using second near-infrared wavelengths
Source: J Biomed Opt. 2025 Feb 18;30(2):026002. doi: 10.1117/1.JBO.30.2.026002 (PMC11833698; doi:10.1117/1.JBO.30.2.026002)
Supplement: Supplementary file 1 [file JBO_030_026002_SD001.pdf]

Supplemental Material for "Photoacoustic Imaging of Rat Kidney Tissue Oxygenation using NIR-II Wavelengths"

Vinoin Devpaul Vincely[1] and Carolyn L. Bayer[1*]
[1]Department of Biomedical Engineering, Tulane University, New Orleans, LA 70112, USA

1. Methods for Monte-Carlo simulations for fluence compensation

Monte Carlo simulations were performed using the MCX toolbox [40]. To model light propagation through a porcine phantom, a computational volume of 1 x 1 x 1.2 cm was defined. A 3 mm thick homogenous slab (dimensions similar to physical sample) was defined and properties were set to that of porcine tissue (Figure S1, retrieved from [56]). A circular Gaussian light beam of 5 mm diameter was defined at z = 0 (illumination surface). The beam divergence was set to 15° (similar to the experimental beam used for sample illumination).

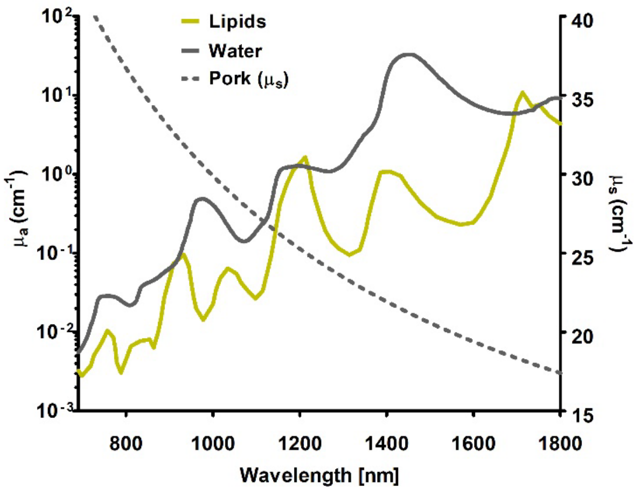

Figure S1: Optical properties used for phantom fluence compensation using MCX. A plot of the optical properties (absorption and scattering) of the porcine slab in Monte-Carlo simulations used for fluence compensation of the acquired photoacoustic images from the phantom experiment (adapted from [56, 53])

To model light propagation through the rat abdomen, a computational volume of 3 x 3 x 3 cm was defined. A mask of the layers of different tissues was developed using a photograph of the sagittal section of the rat abdomen (Fig. S2a, b). Optical properties of the different tissues were set based on values reported in literature [42, 41]. A circular Gaussian light beam of 5 mm diameter was defined at z = 0 (illumination surface). The beam divergence was set to 15° (similar to the experimental beam used for sample illumination).

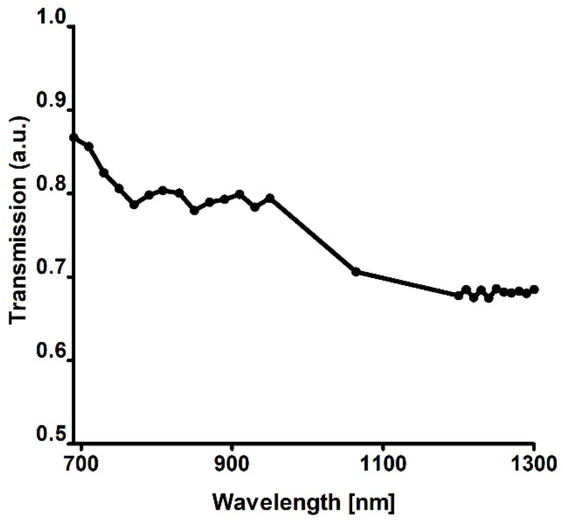

Figure S2: Light attenuation by the transparent (polyethylene terephthalate, PET) sheet. A plot of the transmission of light through the PET sheet used for in vivo rat imaging.

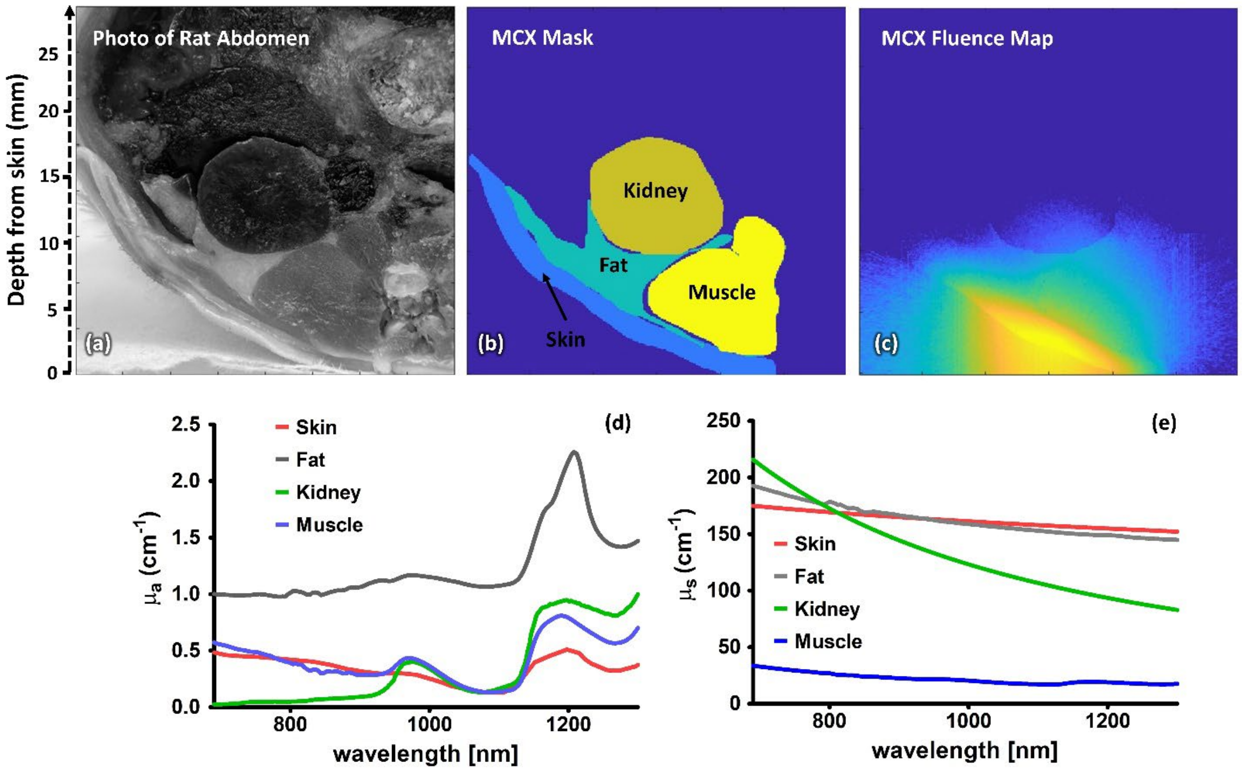

Figure S3: MCX simulations of light propagation through rat abdomen. (a) Photograph of sagittal section of rat abdomen used to generate layer mask. (b) Layer mask used to assign different tissue types for MCX simulations. (c) An example fluence map generated using MCX simulations (properties set at 690 nm). (d-e) Optical properties (absorption & scattering) of the different layer types (skin, fat, muscle & kidney) used for the MCX simulations.

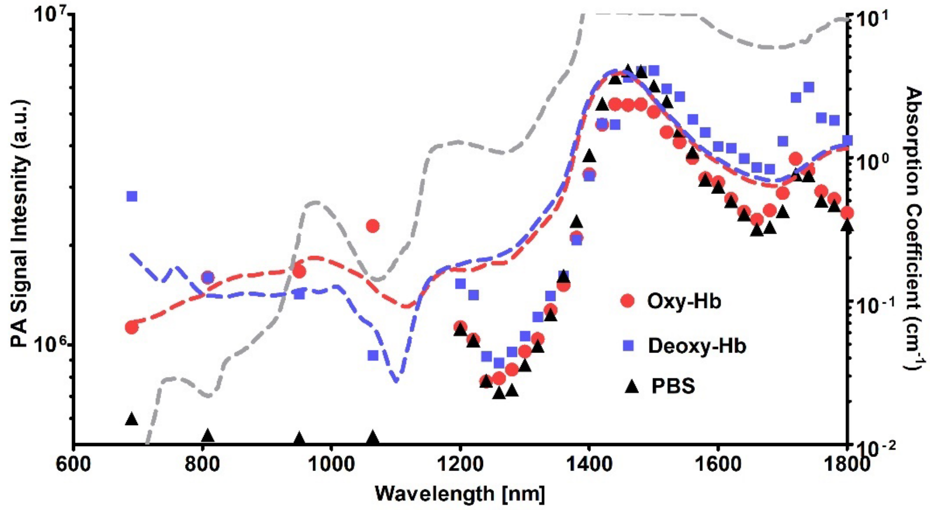

Figure S4: Extended PA spectra of whole blood. PA spectra of whole blood up to 1800 nm – oxygenated (red circles) and deoxygenated (blue squares) and PBS (black triangles) measured in a porcine phantom.

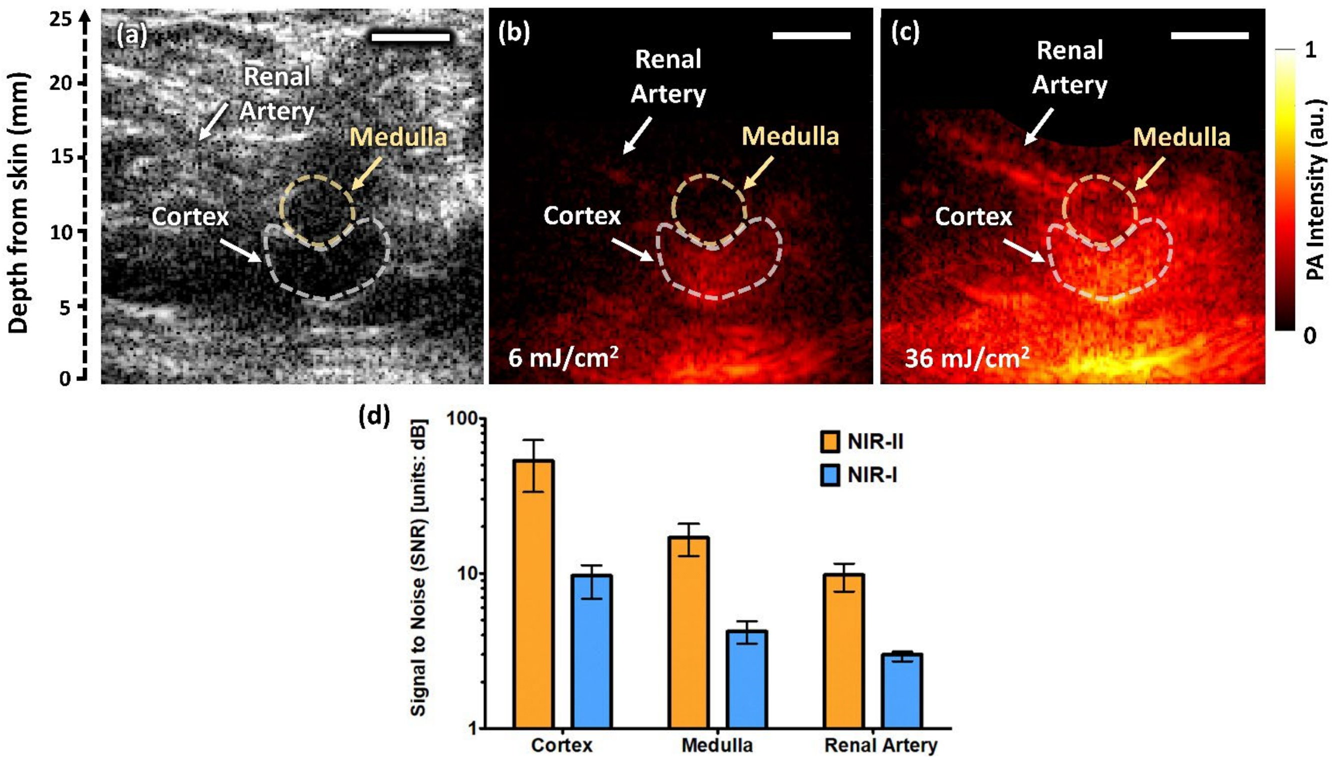

Figure S5: Improvement in signal to noise ratio (SNR) with NIR-II at higher fluences. B-mode (a) and PA images (b-c) of oxygenated rat kidney acquired at 1064 nm with optical fluence at the surface of skin of 6 mJ/cm^2 (b) and 36 mJ/cm^2 (c), respectively. The imaging depth, relative to the skin surface, is shown as the y-axis in (a-c). The scale bars on Fig. S5a-c represent a 5 mm distance. (d) A plot of the signal to noise (SNR) ratio (units of dB) of the different anatomical regions. The SNR is calculated as a ratio between the average PA signal within the individual outlined regions of interest and a region outside the animal body corresponding to system noise. Orange and blue bars correspond to mean SNR at NIR-II and NIR-I, respectively, with error bars representing the standard deviation of five acquisitions.

To demonstrate the higher impact of scattering (μ'_s) over absorption (μ_a) on maximum light penetration depth, MCX simulations were performed to model light propagation through a homogenous layer with varying combinations of absorption (μ_a) and reduced scattering (μ'_s) coefficients (Figure S6). The μ_a and μ'_s values of the sample were varied between 0 – 1 cm^{-1} (15 values) and 0 – 20 cm^{-1} (10 values), respectively. A homogeneous tissue volume of 3 cm × 3 cm × 3 cm was defined and irradiated by a planar source of 1.5 x 1.5 mm. A total of 150 simulations, with varying μ_a and μ'_s values, were performed. The time-averaged fluence of the 2.5 cm deep lateral plane as μ_a and μ'_s are varied is represented by the colormap in Figure S6. For comparison, a generic tissue has corresponding μ_a and μ'_s values represented by the black (690 nm) and red dots (1064 nm). Comparing these two combinations, the 1064 nm combined attenuation provides a roughly 10× increase in fluence vs 690 nm. From these simulations, the impact of the reduced scattering in the NIR-II has a greater influence on the depth of light propagation than the increased attenuation at this wavelength range, when compared to NIR-I.

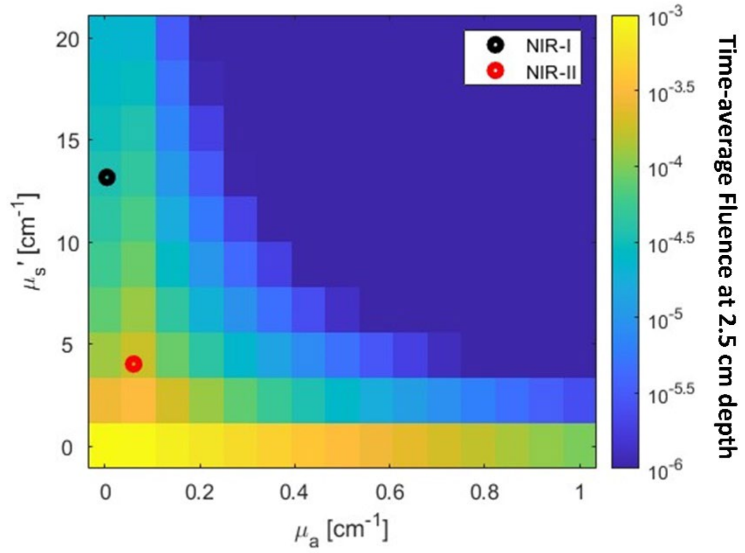

Figure S6: Analysis of relative impact of μ_a and μ'_s from MCX simulations. The average fluence at a depth of 2.5 cm from the surface of illumination for varying permutations of absorption and reduced scattering coefficients is represented by the scaled color bar. The red and black circles correspond to the optical properties of tissue at NIR-I and NIR-II wavelengths, respectively (retrieved from [57]).
